# Genetic and Epigenetic Inheritance at Telomeres

**DOI:** 10.3390/epigenomes6010009

**Published:** 2022-03-16

**Authors:** Evan H. Lister-Shimauchi, Benjamin McCarthy, Michael Lippincott, Shawn Ahmed

**Affiliations:** 1 Department of Genetics, University of North Carolina, Chapel Hill, NC 27599-3280, USA; elistshi@email.unc.edu (E.H.L.-S.); benmcca@med.unc.edu (B.M.); mlippincott@unc.edu (M.L.); 2 Department of Biology, University of North Carolina, Chapel Hill, NC 27599-3280, USA

**Keywords:** telomerase, telomere, POT1, transgenerational, inheritance, non-Mendelian

## Abstract

Transgenerational inheritance can occur at telomeres in distinct contexts. Deficiency for telomerase or telomere-binding proteins in germ cells can result in shortened or lengthened chromosome termini that are transmitted to progeny. In human families, altered telomere lengths can result in stem cell dysfunction or tumor development. Genetic inheritance of altered telomeres as well as mutations that alter telomeres can result in progressive telomere length changes over multiple generations. Telomeres of yeast can modulate the epigenetic state of subtelomeric genes in a manner that is mitotically heritable, and the effects of telomeres on subtelomeric gene expression may be relevant to senescence or other human adult-onset disorders. Recently, two novel epigenetic states were shown to occur at *C. elegans* telomeres, where very low or high levels of telomeric protein foci can be inherited for multiple generations through a process that is regulated by histone methylation.Together, these observations illustrate that information relevant to telomere biology can be inherited via genetic and epigenetic mechanisms, although the broad impact of epigenetic inheritance to human biology remains unclear.

## 1. Introduction

**Figure 1:**
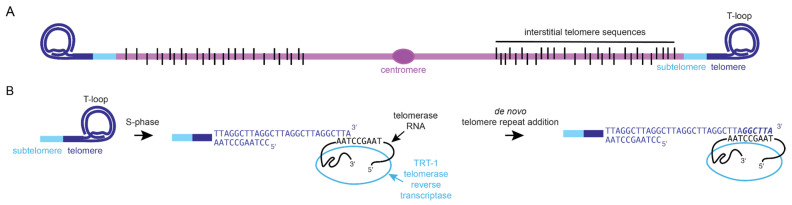
Telomeres and telomerase. (**A**) Telomeres typically end in tandem repeats and a 3′ single-stranded overhang that intercalates into double-stranded telomeric DNA. Black bars represent interstitial telomere sequences, which are tracts of degenerate telomere repeats scattered along chromosome arms. (**B**) The T-loop can unfold to reveal a 3′ single-stranded overhang that allows for de novo telomere repeat addition by *C. elegans* telomerase. The template for telomere repeat biogenesis is encoded by a hypothetical *C. elegans* telomerase RNA subunit.

The termini of linear chromosomes are composed of DNA–protein complexes called telomeres. Telomeres are required to promote chromosome stability by preventing the loss of terminal DNA sequence during cell replication and by preventing chromosome ends from being recognized as inappropriate DNA damage that needs to be repaired. Telomeric DNA is typically composed of tracts of simple repetitive DNA sequences, such as (TTAGGG)_n_ in mammals, (TTAGGC)_n_ in nematodes, and (TTGGGG)_n_ in *T. thermophila*. Telomeres end with a 3′ overhang that is 10 to 50 nucleotides in length, whereas double-stranded telomeric DNA ranges dramatically in length from tens to thousands of base pairs depending on the species. The repetitive nature of telomeric DNA allows the 3′ overhang to fold back and form a strand invasion intermediate within double-stranded telomeric DNA. This lariat-like structure, termed a T-loop, protects chromosome termini from being recognized as DNA damage (Figure 1A) [1,2].

Human somatic cells can be cultured for 40–60 population doublings before entering an irreversible state of cell cycle arrest termed senescence [3]. This hallmark of human cell biology, known as the Hayflick limit, was linked to aging by a study that demonstrated that cells isolated from neonates can proliferate for many more divisions than cells isolated from elderly individuals [4]. To explain this link between somatic cell proliferation and aging, it was hypothesized that a small amount of terminal DNA sequence might be lost during each round of DNA replication, in part due to short RNA primers that initiate DNA replication [5]. In an initial step towards addressing this hurdle, known as the “end replication problem” [6,7], telomeres were identified as a simple repetitive sequence based on analysis of a tiny rDNA chromosome that is abundant in the macronucleus of *Tetrahymena thermophila* [8]. *Tetrahymena* possesses a germline micronucleus with five chromosomes that develops via chromatin diminution into a somatic macronucleus with ~200 chromosomes that are amplified to create ~18,000 somatic chromosomes [9,10]. As the large number of macronuclear telomeres might shed light on the end replication problem, Blackburn and Greider focused on *Tetrahymena* cell extracts, where they identified a ribonucleoprotein, telomerase, that can add telomere repeats to (TTGGGG)_n_ oligonucleotides that mimic the *Tetrahymena* telomeric overhang [11,12]. Identification of telomerase RNAs from ciliates and mammals revealed that they contain a template sequence that is reverse transcribed onto the chromosome termini (Figure 1B) [13,14,15,16].

The existence of telomerase supported the idea that terminal DNA loss during cell division might be relevant to the Hayflick limit. Human somatic cells were shown to display telomere erosion as they proliferate in vitro and to be deficient for telomerase activity [17,18]. The telomerase reverse transcriptase (TERT) subunit was not expressed in human somatic cells, and TERT expression was sufficient to restore in vitro telomerase activity and cellular immortality to human fibroblasts [19,20]. Senescent human fibroblasts were found to possess DNA damage foci that colocalize with telomeres and enforce cell cycle arrest [21]. Furthermore, DNA damage foci were associated with telomeres in cells of aged but not young primates [22], indicating that telomere-induced senescence or “the telomere clock" is relevant to aging. Together, these experiments provided firm evidence that telomere erosion explains the Hayflick limit [23].

Mammalian telomere-binding proteins TRF1 and TRF2 interact with telomeric double-stranded DNA and are associated with single-stranded telomere-binding protein POT1, which interacts with the 3’ overhang and cofactors TPP1, TIN2, and RAP1. Together, these proteins form a complex termed shelterin, which promotes telomere integrity and function [24]. TRF2 promotes T-loop formation [25,26], and dysfunction of TRF2 is sufficient to trigger senescence in human cells with long telomeres [27]. The ability of telomere-binding proteins to suppress DNA repair at chromosome termini, which are normal DNA double-strand breaks, may contribute to aging independent of telomere length because DNA damage near telomeres may be irreparable and is capable of triggering senescence [28]. These observations support the view that short dysfunctional telomeres as well as longer telomeres associated with DNA damage are likely to induce senescence during human aging.

Although somatic telomere erosion and other stresses may contribute to cellular aging, a beneficial effect of senescence is to limit inappropriate cellular proliferation [29]. The relevance of senescence as a tumor suppressor mechanism is apparent in human cancer cells, most of which express telomerase. Among the most recurrent mutations in human tumors are activating mutations in the TERT promoter [30,31,32,33]. The remaining ~10% of tumors that lack telomerase maintain their telomeres via a recombination-based telomere maintenance process termed alternative lengthening of telomeres (ALT) [34].

Silencing of telomerase in somatic cells evolved on at least four independent occasions in the course of mammalian evolution [35,36,37,38]. Larger mammals, such as primates, whales, bears, and elephants, have vast numbers of somatic cells to control and benefit from silencing of somatic telomerase, which creates a powerful tumor suppressor mechanism that is known as the “telomere clock”. Species that silence somatic telomerase, such as primates, often possess shorter telomeres (5–15 kb) compared to smaller mammals that express somatic telomerase, such as mice or squirrels (25–50 kb) [35,36].

Although telomere length does not correlate with mammalian longevity, the rate of telomere erosion is slower in long-lived mammals [39,40]. Therefore, silencing of somatic telomerase may not be the only variable that is relevant to mammalian development. Consistently, the protein TRF2 is required to promote T-loop formation in mouse somatic cells but was recently shown to be dispensable for telomere stability in embryonic stem cells [41]. Telomerase is primarily active during human embryonic development, particularly around the time of blastulation [42], but is much lower or absent in somatic cells, including stem cells [43].

The cost of somatic telomere shortening is suggested by rapid telomere attrition in cells of humans who suffer from the early-onset aging disorders Werner syndrome and Hutchinson–Gilford progeria. Werner syndrome is an adult-onset disorder caused by a mutation in the DNA helicase WRN, whereas Hutchinson–Gilford progeria presents in early childhood and is caused by a mutation of lamin A, which coats the inner nuclear envelope and physically interacts with telomeres [44,45,46]. A mechanistic link between these disorders is suggested by atypical Werner syndrome, which can be caused by lamin A mutations [47]. Although mice that are mutant for WRN have normal life spans [48], *wrn* mutant mice that are also deficient for telomerase display rapid telomere erosion and age prematurely [49]. Therefore, although the telomere clock has beneficial impacts for large mammals, including tumor suppression, this clock becomes costly in certain developmental settings [50].

Each person inherits a stochastic number of long, moderate, or short telomeres - 46 per parental gamete - as well as parental chromosomes that possess genome polymorphisms that may modulate telomere length during embryogenesis [51]. Genotypically wild-type children of parents who are deficient for telomerase can display either normal or short telomere lengths [52,53], whereas two generations of wild-type telomerase activity restores telomere length [54]. However, telomerase RNA mutant mice can give rise to genotypically wild-type animals that have either normal or short telomeres, and those with short telomeres can transmit short telomeres for at least six generations [55]. Although human telomere length may be a highly heritable trait [55,56], future efforts that utilize long-read genome sequencing technologies may help to more comprehensively characterize how individual human telomeres are transmitted from parent to child.

The telomere clock is not simply set at conception based on gametes that deliver telomeres of specific lengths in conjunction with genome polymorphisms that regulate telomere length. Environmental stimuli can affect human telomeres throughout life. For example, humans who experience psychological stress display shortened leukocyte telomeres, which indicates that telomere length in blood cells can be epigenetically modulated in a manner that may be relevant to senescence or longevity [57,58]. Stressed mothers not only display shorter leukocyte telomeres but also transmit shorter telomeres to their babies, emphasizing the systemic nature of the response of telomeres to social stress [59]. On the other hand, leukocyte telomere length can increase in response to activities such as exercise, yoga, and even space travel [60,61,62,63].

In this review, we present a framework for considering genetic and epigenetic aspects of telomere biology that may be relevant to inheritance.

## 2. Genetic Inheritance of Mammalian Telomeres and Their Health Impact

Analysis of the 3′ end of human telomerase RNA revealed a Box H/ACA domain that promotes the stability of noncoding nucleolar RNAs [64]. Box H/ACA domain RNAs interact with the yeast protein Cbf5p, whose human homolog DKC1 causes dyskeratosis congenita, an X-linked genetic disorder that affects highly proliferative tissues of the skin and bone marrow. Cells from dyskeratosis congenita patients were shown to possess low levels of telomerase and shortened telomeres [64]. Although DKC1 is a pseudouridine synthase that promotes biogenesis of various noncoding nucleolar RNAs, some of which guide rRNA maturation [65], DKC1 mutations that cause dyskeratosis congenita preferentially compromise telomerase RNA stability. Proof that telomerase dysfunction causes bone marrow failure was revealed by mutations in the RNA subunit of telomerase in several families that transmit an autosomal dominant form of dyskeratosis congenita [66]. Telomerase RNA mutations were also found in individuals who suffer from aplastic anemia in the absence of skin abnormalities observed in dyskeratosis congenita [67]. The pathological consequences of short telomeres were further broadened when telomerase mutations were identified in families whose members had idiopathic pulmonary fibrosis, most of whom lacked features of dyskeratosis congenita [53,68].

Telomerase dysfunction and shortened telomeres are hallmarks of a range of clinical disorders that impair stem cell function [69]. The majority of human disorders associated with telomere erosion are multigenerational and are thought to initiate with a spontaneous mutation in a parental germ cell. Individuals who inherit a mutation that compromises telomerase possess shortened telomeres and can display mild phenotypes in late life. Children and grandchildren who inherit shortened parental telomeres and a parental mutation that compromises telomerase activity will display a range of phenotypes that depend on telomere length. Individuals with mutations that compromise telomerase and have short telomeres develop pulmonary fibrosis in their 50s or 60s, those with very short telomeres commonly develop aplastic anemia in their 20s or 30s, and those with even shorter telomeres develop dyskeratosis congenita that affects school-age children. Hoyeraal–Hreidarsson syndrome affects children under five and almost never occurs in a multigenerational context [69,70]. Multigenerational families commonly transmit mutations in reverse transcriptase or noncoding RNA subunits of telomerase or in distinct proteins that normally promote telomere repeat addition by telomerase in germ cells [71].

Shortening of telomeres over multiple generations can also occur in response to deficiency for the antirecombinase RTEL1 [72], which is an inhibitor of homologous recombination that normally dismantles T-loops. RTEL1 also dismantles stable secondary structures called guanine quadruplexes, which can form when telomeric DNA is replicated [73]. Transgenerational telomere erosion can also occur in response to defects in the CST1–STN1–TEN1 (CST) complex, which interacts with single-stranded telomeric overhangs created by telomerase and recruits DNA polymerase and primase to convert most of the telomeric 3’ overhang into double-stranded telomeric DNA [74,75].

Very young individuals can display severe forms of dyskeratosis congenita termed Hoyeraal–Hreidarsson and Revesz syndromes, which are associated with developmental delay and neurological defects [76]. These early-onset telomere disorders can be caused by biallelic mutations in TERT, which elicit a strong telomerase defect accompanied by shortened telomeres inherited from both parents or by biallelic mutations in RTEL1 [77,78,79,80]. Hoyeraal–Hreidarsson syndrome can also be caused by DKC1 mutations that affect biogenesis of telomerase RNA and rRNA [70,81]. We conclude that mutations in human RTEL1 or DKC1 can have gradual multigenerational or acute single-generation health consequences. 

Mouse telomerase RNA mutants display multigenerational telomere erosion accompanied by defects reminiscent of premature aging, including impaired hematopoietic and lymphocyte proliferation, decreased body weight, hair graying, and alopecia [82,83]. Shortened telomeres in these telomerase RNA mutants also induce chromosome instability and tumor formation [83,84]. This is consistent with a modest increase in cancer incidence in families that transmit shortened telomeres [85].

Idiopathic pulmonary fibrosis is a rare disorder that occurs spontaneously in older humans, half of whom have telomeres that rank in the lowest 1% of their age group [86]. While downregulation of telomerase activity can lead to disease, upregulation can also have negative consequences. Variants in the human genome that are associated with long telomeres confer a higher risk for certain types of cancer, including melanoma, lung cancer, glioma, and ovarian cancer, whereas other noncommunicable diseases, including cardiovascular, autoimmune, and neurological disorders, are unaffected [87,88,89]. Therefore, delay in the telomere clock that induces senescence promotes the development of some types of cancer. Excessive proliferation of somatic cells might allow mutations in tumor suppressors or oncogenes to accumulate, leading to cancer development. Alternatively, even though senescent cells promote aging in mice [90], accumulation of senescent cells in aging humans may result in a cell-nonautonomous activity that suppresses tumor development [29]. It is also possible that moderate to short telomere lengths result in an epigenetic effect on gene expression at human subtelomeres or elsewhere in the genome that suppresses tumor development [91].

## 3. Epigenetics of Telomeres

Classic studies have revealed that a gene placed next to an *S. cerevisiae* telomere displays a heritable on/off transcriptional state that is revealed in sectoring yeast colonies [92,93]. This defining hallmark of heterochromatin is known as the telomere position effect and is potentiated by long telomeres [94], the N-terminal tails of histones H3 and H4, as well as three silent information repressor proteins Sir2, Sir3, and Sir4 that normally enforce genome silencing at the mating-type locus [95]. Analysis of stress-resistant yeast mother cells revealed a dominant mutation in the *sir4* gene that induces longevity [96]. This separation-of-function mutation, *sir4-42*, caused the SIR silencing complex to relocalize from telomeres to the nucleolus [96,97], where it suppressed accumulation of toxic extrachromosomal rDNA circles [98]. Together these studies led to an elegant model that telomeres harbor silencing factors that are capable of silencing nearby genes [99] but that the Sir2/3/4 silencing complex can translocate to the nucleolus to promote rDNA stability and longevity in *S. cerevisiae* mother cells [100].

A thorough study of native *S. cerevisiae* telomeres found that many genes that are normally adjacent to telomeres display little transcriptional repression [101]. In *S. cerevisiae*, X elements are found at most telomeres, and Y′ elements are adjacent to half of telomeres. Silencing of genes near several *S. cerevisiae* subtelomeres depends on their proximity to an X element, which implies that telomeres may not be sufficient to induce a position effect [102]. Furthermore, genome-wide mapping efforts revealed that SIR silencing factors can be associated with loci that are not silent [103]. In addition, roughly half of Y′ elements are transcribed despite being adjacent to telomeres [104,105]. Genome-wide analysis of Sir2, Sir3, and Sir4 silencing factors and their effects on transcription revealed that these proteins are not enriched at most telomeres and that they repress only six percent of naturally occurring subtelomeric genes [105]. As half of the 42 genes silenced by Sir proteins in *S. cerevisiae* are subtelomeric, telomeres occasionally repress subtelomeric gene transcription, but this silencing does not spread far from telomeres and is not relevant at many telomeres.

*Drosophila* telomeres are heterochromatic in nature and can exert a position effect that silences nearby genes [106], reminiscent of the telomere position effect observed in *S. cerevisiae* [92]. Unlike most organisms, *Drosophila* telomeres are composed of non-LTR retrotransposons [107], which are genomic parasites that are silenced by small RNA-mediated heterochromatin formation [108].

Telomeric and subtelomeric DNA has been reported to be enriched for heterochromatic marks in mammalian cells [109,110,111,112,113]. This concept is supported by studies of mouse cells deficient for two methyltransferases that promote heterochromatin formation, Suv39h and Suv4-20h, which display elongated telomeres characteristic of ALT cells [109,114]. Telomeric chromatin is likely to be altered in human tumors that rely on ALT, most of which are mutant for the histone chaperone ATRX/DAXX that deposits H3.3 in silent regions of the genome, including pericentromeres and tandem repeats [115,116]. Furthermore, histone H3.3 G34R mutations and H3.3 K27M mutations are found in a subset of ALT tumors. The chromatin defect that results from loss of ATRX/DAXX or altered histone H3.3 may promote the recombination process that creates long and heterogeneous telomeres in ALT tumors [34]. ALT cells may possess telomeres with decreased levels of H3K9me3 or H3K27me3 silencing marks that result in a less compact euchromatic state that is amenable to recombination [117].

**Figure 2:**
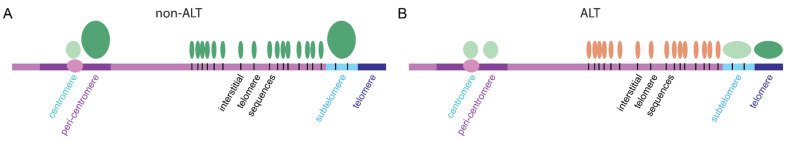
Models of heterochromatin at human pericentromeres, interstitial telomere sequences, subtelomeres, and telomeres in cells that use non-ALT or ALT telomere maintenance mechanisms. Dark green ovals indicate heterochromatin, large amounts of which are present at pericentromeres and at many subtelomeres in non-ALT cells (**A**). In ALT cells that are dysfunctional for ATRX/DAXX, pericentromeric silencing is disrupted, subtelomeric silencing is inconsistently affected (light green), and telomeres possess H3K9me3 (**B**). Interstitial telomere sequences normally form small blocks of heterochromatin whose status in ALT cells is not clear (orange).

Studies of the epigenetic state of telomeric chromatin can be complicated by interstitial telomere sequence (ITS) tracts, which are stretches of degenerate telomere repeats scattered along chromosome arms. Large vertebrate ITS tracts can be found in heterochromatic segments of the genome, such as the pericentromere [118]. Studies of telomeric chromatin have traditionally assessed the presence of telomeric DNA based on ChIP assays or nuclease sensitivity assays where blotted DNA is detected using a telomeric probe that can recognize both telomeres and degenerate ITS tracts (Figure 2). To eliminate the confounding effects heterochromatic ITSs may have on ChIP hybridization assays, ChIP-seq was used to study the chromatin status of telomeres in a variety of cell lines and tumor samples. This revealed that telomeres of non-ALT human cell lines have low levels of the H3K9me3 heterochromatin mark and, surprisingly, high levels of euchromatic marks H3K27ac and H4K20me1, whereas ALT telomeres possess high levels of H3K9me3 [112,119]. An increased level of the H3K9me3 heterochromatin mark at ALT telomeres could reflect the release of heterochromatin factors that are normally deposited by ATRX/DAXX at heterochromatic segments of the genome (Figure 2).

ChIP-seq analyses have revealed that telomeric DNA is enriched for the heterochromatin mark H3K9me2 in *C. elegans* [120,121,122] and H3K9me2 and H3K27me3 in *Arabidopsis* [123,124]. Studies of mammalian telomeric chromatin have generally focused on the heterochromatic marks H3K9me3, H3K20me3, and H3K27me3, so it remains possible that the H3K9me2 mark is present at normal mammalian telomeres and that loss of ATRX/DAXX in ALT tumors results in transition to an H3K9me3 heterochromatic state [112,119]. If mammalian telomeres are normally euchromatic, which may be possible given the ChIP-seq data discussed in the previous paragraph, then subtelomeric DNA that is repetitive in nature and heterochromatic might contribute to silencing of some subtelomeric genes (Figure 2) [125,126]. The epigenetic state of mammalian telomeres may be more thoroughly characterized in future ChIP-seq studies.

Long telomeres can stimulate silencing of a subtelomeric luciferase transgene in human cells [127]. A subsequent study revealed that telomere erosion in primary human fibroblasts or myoblasts is consistently accompanied by altered expression of 15 genes that were within 10 Mb of three telomeres (1p, 6p, and 12p), many of which displayed consistent increases in expression in response to telomere shortening [91]. Therefore, human subtelomeric gene expression can be modulated by telomere length. Subtelomeric silencing is thought to play a causal role in the autosomal dominant human disorder facioscapulohumeral muscular dystrophy (FSHD). This adult-onset disorder is associated with expression of the DUX4 gene, which is immediately adjacent to the telomere of chromosome 4q [128]. The DUX4 gene is typically associated with an array of ~100 D4Z4 satellite repeats that promote genomic silencing of DUX4. However, FSHD can occur when the D4Z4 repeat copy number is reduced to less than 10. If primary cells from FSHD patients are isolated and telomere length is homogenized by transient expression of telomerase reverse transcriptase, this induces silencing of DUX4. Proliferation of these cells in the absence of telomerase results in accumulation of shortened telomeres characteristic of young or middle-aged adults, which induces transcriptional upregulation of DUX4 and several adjacent genes [129]. These data indicate that FSHD and subtelomeric DUX4 gene expression is an epigenetic consequence of at least two changes to the genome, namely shortening of D4Z4 satellite repeats and shortening of telomeres, both of which can be transmitted by parental gametes. About 1% of humans have <10 D4Z4 repeats, but very few of these individuals get FSHD. One plausible explanation for this discrepancy is stochastic variation in 4q telomere length may contribute to development of FSHD.

A second muscular dystrophy that may interact with telomere length is Duchenne muscular dystrophy, which is an X-linked disorder in the *DYSTROPHIN* gene that affects 1 in 3500 males. Loss of *DYSTROPHIN* typically results in childhood weakness, and disease symptoms worsen progressively over two or three decades of life as a consequence of premature senescence of muscle stem cells that proliferate excessively [130]. Mouse *DYSTROPHIN* mutants only exhibit mild symptoms [131,132]. Given that laboratory mouse strains have long telomeres (>25 Kb) [133], mouse double mutants that lack both *DYSTROPHIN* and the telomerase RNA subunit *mTR* were constructed. This resulted in second-generation mice that possess short telomeres and penetrant skeletal muscle and cardiac defects analogous to those observed in Duchenne muscular dystrophy, which supports a role for the epigenetic consequences of shortened telomeres in triggering Duchenne muscular dystrophy [134]. If natural telomere erosion in human somatic cells triggers epigenetic changes that are relevant to muscular dystrophy, comprehensive analysis of subtelomeric gene expression in response to telomere erosion may be warranted.

Telomeric chromatin state can affect the expression of telomeric-repeat-containing RNA (TERRA), which are RNA transcripts that extend into telomeric repeats. Stress in mice can lead to the phosphorylation of ATF7, a chromatin regulator, causing it to release from TERRA promoters [135]. Phosphorylation of ATF7 in germ cells leads to TERRA upregulation in sperm, resulting in shortened telomeres in subsequent progeny [135]. If levels of TERRA in germ cells modulate telomere length in future generations, then transcription of this short noncoding RNA may contribute to genetic or epigenetic inheritance at telomeres.

## 4. Transgenerational Epigenetic Inheritance

The telomere position effect is an elegant example of an epigenetic on/off state in gene expression that is mitotically transmitted. Transgenerational epigenetic inheritance concerns meiotic transmission of information that does not involve a DNA sequence change. Mediators of transgenerational epigenetic inheritance include loci that are marked by altered histone modifications or DNA methylation as well as small RNAs that guide epigenetic modifications. Other epigenetic factors that gametes can transmit include metabolites or yolk that might modify development or aging in future generations [136].

Transgenerational epigenetic inheritance can occur in response to an environmental stress and results in modified germ cells that are transmitted for one or several generations. Such a mechanism might be adaptive if it improves fitness by enhancing the ability of progeny to withstand stress. In humans, transgenerational epigenetic inheritance has been observed in response to two historic famines, where embryos in utero at the time of famine mature into parents who give rise to children and grandchildren with increased frequencies of type 2 diabetes, metabolic syndrome, and hypertension [137,138]. These pathological conditions may not appear beneficial but might create metabolic changes that would promote survival if famine were to recur. Deficiency for dietary compounds such as vitamin D in mothers is associated with intergenerational cardiometabolic and neuronal defects in children [139].

Due to its potential relevance to humans, the effects of diet on epigenetic inheritance have been studied in mice. Excessive caloric intake by male but not female rats can lead to dysfunction in insulin-producing beta cells in progeny, an effect that may be facilitated by cytosine methylation [140]. Male mice fed a low protein diet sire progeny with altered gene expression and metabolism, where inheritance may be modulated by tRNA fragments transported into developing spermatozoa via vesicles secreted from the epididymis [141,142]. The transmission of RNA from soma to developing spermatozoa may be essential for embryonic development, emphasizing the importance of intergenerational information transfer as a mechanism that may integrate physiology with the environment [143]. Other studies have found that pathological heritable and sometimes transgenerational epigenetic effects, including obesity, can result from exposure of mice to man-made chemicals, such as DDT, BPA, and components of jet fuel [144,145]. While intriguing, how much inheritance in humans or mammals is explained by epigenetic inheritance remains an unsolved question in experimental biology.

## 5. Transgenerational Epigenetic Inheritance at *C. elegans* Telomeres

Although epigenetic analysis of telomeres has been studied in some detail, a single study was recently published on the topic of transgenerational epigenetic inheritance at telomeres [146]. Deficiency for two *C. elegans* shelterin proteins is sufficient to create gametes that induce high or low levels of telomeric foci composed of these proteins for several generations.

**Figure 3:**
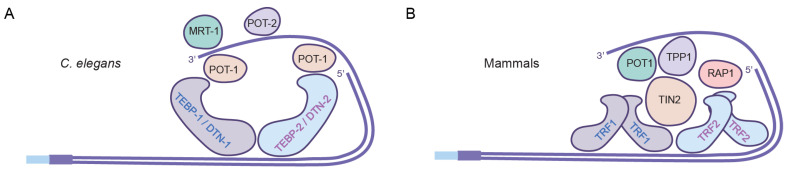
Telomere proteins in *C. elegans* (**A**) and mammals (**B**).

**Figure 4:**
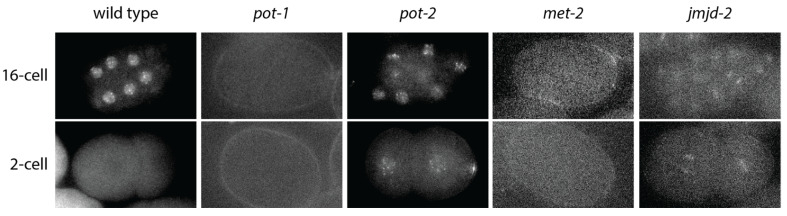
Telomeric foci in *C. elegans* embryos. Low levels of telomeric foci are present in two-cell wild-type embryos, but high levels are observed in 16-cell embryos. The progeny of *pot-1* and *met-2* mutants display constitutively low levels of telomeric foci, whereas the progeny of *pot-2* and *jmjd-2* mutants display constitutively high levels of telomeric foci.

The mammalian single-stranded DNA-binding protein POT1 has three homologs in *C. elegans*: POT-1 and POT-2, which repress telomerase activity at telomeres, and MRT-1, which is required for telomerase activity at telomeres (Figure 3A) [147,148]. POT-1 and POT-2 form nuclear foci at telomeres. Analysis of POT-1::mCherry revealed that, on average, one nuclear focus is present per telomere in pachytene meiotic germ cells [147]. Although strong telomeric foci are present in adult sperm, they vanish in one-cell embryos and then gradually increase during embryonic development (Figure 4). Mutation of *pot-2* results in high levels of POT-1::mCherry telomeric foci at all stages of embryonic development, whereas mutation of a second component of telomeric foci, *pot-1*, results in elimination of mNeonGreen::POT-2 telomeric foci from almost all nuclei at all developmental stages (Figure 4) [146].

Surprisingly, *C. elegans* germ cells transmit an epigenetic memory of the low or high levels of telomeric foci present in *pot-1* or *pot-2* mutants, respectively. Gametes of *pot-1* mutants give rise to progeny with low levels of telomeric foci for two generations, which is formally termed intergenerational inheritance. Progeny of *pot-2* mutants display a more potent memory, where high levels of telomeric foci can persist for six generations, even for F2 progeny where homozygous wild-type *pot-2*(+) gene expression has been restored [146]. Either sperm or oocytes of *pot* mutants can alter levels of telomeric foci for multiple generations of cross-progeny, which suggests a form of nuclear inheritance [149].

Small RNA pathways can alter gene expression for multiple generations [150,151], and small RNAs with homology to telomeres have been identified in *C. elegans* and other species [120,123,152,153]. Therefore, six mutations that compromise endogenous small RNA pathways were tested for effects on telomeric foci, including *dcr-1*/Dicer and *prg-1*/Piwi, but wild-type levels of telomeric foci were observed [146]. The lack of a role for small RNAs suggests that another epigenetic factor alters levels of telomeric foci in future generations.

Classical experiments in *S. cerevisiae* revealed that telomeres can epigenetically silence subtelomeric gene expression in a manner that is mitotically heritable and reversible [92]. Mutation of three histone methyltransferases that result in genomic silencing defects, *met-2*, *set-25*, and *set-32*, [122,154], were found to create progeny with very low levels of telomeric foci. Conversely, mutation of the H3K9 demethylase *jmjd-2*, which results in excessive levels of H3K9 methylation and genome silencing, led to gametes whose progeny possessed constitutively high levels of telomeric foci, similar to progeny of *pot-2* mutants (Figure 4) [146]. As MET-2, SET-25, and SET-32 promote heritable genomic silencing by methylating H3K9 and H3K23 in response to small RNAs [154,155], these histone methyltransferases may be recruited to telomeres by a sequence-specific DNA-binding protein, such as POT-1.

In wild-type animals, sperm that possess bright telomeric foci fertilize oocytes with diminished levels of telomeric foci to create one-cell embryos whose pronuclei lack telomeric foci. Although telomeric foci are transiently eliminated, they reappear in two- or four-cell wild-type embryos, and then the number of foci per nucleus rapidly increases [146]. In contrast, when a *pot-2* mutant sperm fertilizes a wild-type oocyte, the wild-type pronucleus of one-cell zygote immediately displays high levels of telomeric foci. This suggests a potent diffusible factor that emanates from a *pot-2* mutant pronucleus that immediately suppresses loss of telomeric foci. Because cross-progeny of *pot-1*; *pot-2* double mutants have very low levels of telomeric foci, similar to *pot-1* single mutants, the POT-2 single-stranded DNA-binding protein or a factor it modulates may alter levels of telomeric foci via a process that is heritable and whose memory persists for multiple generations.

## 6. Future Directions

Although *pot-2* mutant gametes rapidly induce high levels of telomeric foci in embryos, it takes a number of generations to recover to the wild-type state, where telomeric foci are eliminated in one-cell embryos. This gradual multigenerational recovery is consistent with what has been observed in five examples of transgenerational epigenetic inheritance in *C. elegans* [149,156,157]. First, expression of a *C. elegans* transgene can be modulated by heat stress for 10 generations [158] via a silencing process that requires the H3K9 methyltransferases MET-2 and SET-25, which are required for creation of stable telomeric foci. Second, starvation of L1 larvae for eight days and maintenance of the stress-resistant dauer state for multiple weeks results in transgenerational transmission of traits that influence growth rate, reproduction, stress resistance, and adult longevity [157,159]. Third, if *C. elegans* is grown for successive generations in the presence of the mitochondrial poison antimycin A, it induces a resistance to this poison that persists for three generations by altering expression of mitochondrial stress response genes [156]. Fourth, exposure to heavy metals or oxidative stress increases resistance to similar stress in subsequent generations in *C. elegans*, in part through H3K4 trimethylation [160]. Fifth, deficiency for the SET-2/ASH-2/WDR-5 trithorax complex induces longevity in a manner that is inherited for three generations [161,162]. Although levels of telomeric foci were not altered in progeny of set-2 mutants [146], it is possible that another example of transgenerational epigenetic inheritance might employ altered levels of telomeric foci as a mechanism of transgenerational memory.

Studies in model organisms have revealed many epigenetic and genetic effects that persist for two or more generations. However, single-generation effects are easier to study, especially in humans, and are often more pronounced because the intensity of transgenerational phenotypes can diminish in later generations. Analysis of telomere length in human families with telomerase mutations has created strong models of transgenerational genetic inheritance of shortened telomeres that can persist for multiple generations. While telomere erosion is a genetic change, shortened telomeres can have epigenetic relevance to disease, as suggested by studies of two muscular dystrophies [129,134].

Telomerase is silenced in the somatic cells of many large mammals, and the double-stranded DNA telomere-binding protein TRF2 is required for T-loop formation in differentiated mouse fibroblasts but not in embryonic stem cells [41]. These dynamic aspects of mammalian telomere biology suggest that shelterin proteins might differentially localize to telomeres during development, as observed for *C. elegans* POT-1 and POT-2. If telomeric foci represent heterochromatin domains within the nucleus, then they could serve as reservoirs for heterochromatin factors that could have broad or focused effects on other segments of the epigenome. In addition, proteins that associate with telomeres could have nontelomeric functions that are regulated by an altered epigenetic state at telomeres, as established for the gain-of-function Sir4-42 protein, which promotes mother cell longevity, and for the RAP1 transcription factor, which is associated with telomeres in yeast and mammals [163,164].

Given that null mutations in POT1 compromise fertility in species that possess a single POT1 gene, such as *S. pombe* or the moss *P. patens* [165,166], it may be easier to explore the potential relevance of POT1 to regulation of telomeric foci in species that possess multiple POT1 proteins. The *Arabidopsis* model system has two well-characterized POT1 genes, *POT1a* and *POT1b*, which are important for stimulation of telomerase activity and for chromosome end protection, respectively [167,168,169,170], as well as *POT1c*, which has no known telomeric functions [171]. Humans possess four alternative splice forms of POT1, in addition to a full-length transcript encoding functional POT1 protein, whose expression and function may be worth considering in the context of epigenetic memory [172].

Mice deficient for *RAP1* and *POT1b* telomere proteins are viable. *RAP1*-deficient mice become obese but are typically created from crosses between *RAP1* heterozygotes, so it is not clear if RAP1 homozygous mutant mice transmit a phenotype such as obesity to heterozygous cross progeny [173,174]. Mice have two copies of *POT1*. *POT1a* is essential for mouse development, and its loss induces telomere recombination and elongation as well as senescence [175]. *POT1b* mutant mice are viable, but males experience increased apoptosis and eventually become infertile due to limited sperm production [176]. Loss of either *POT1a* or *POT1b* does not affect association of TRF1, TRF2, or RAP1 with mouse telomeres [177]. To address the potential role for mouse shelterin proteins in transgenerational inheritance, *RAP1* or *POT1b* mutant mice could be crossed with wild-type mice to give rise to heterozygous *RAP1* or *POT1b* progeny that might display altered levels of telomeric foci. If so, this would create a mammalian model system in which altered levels of telomeric foci could be studied for potential effects on mammalian physiology.

Deletion of most mouse shelterin components results in embryonic lethality [178,179,180,181]. Deletion of TRF2 results in mouse embryonic fibroblasts whose telomeres have POT1, TPP1, TIN2, and TRF1 but lack the TRF2-interacting protein RAP1 [182], whereas simultaneous deletion of double-stranded DNA telomere-binding proteins TRF1 and TRF2 results in the loss of all four remaining shelterin subunits from telomeres, leaving telomeric nucleosomes intact [183]. Conditional deletion of essential shelterin proteins in the mouse germ line might result in fertile germ cells that are sufficient to alter levels of telomeric foci for one or more generations, as observed for progeny of *pot-1* and *pot-2* mutants in *C. elegans*.

**Figure 5:**
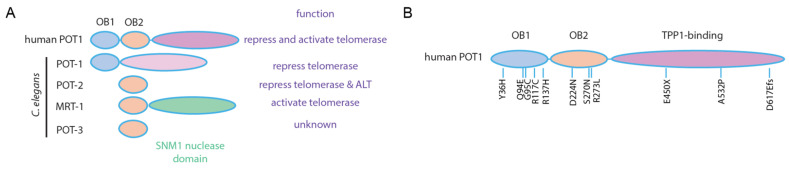
POT1 protein models. (**A**) Human and *C. elegans* POT1 proteins. (**B**) A variety of human germline mutations in *POT1* have been linked to tumor development.

From the perspective of human genetics, somatic and germline mutations in POT1 have been associated with an increased risk for various cancers, including chronic lymphocytic leukemia, Hodgkin lymphoma, colorectal, glioma, melanoma, sarcoma, and angiosarcoma [184,185,186,187,188,189]. Many *POT1* mutations implicated in cancer affect the first and second OB folds of POT1, which are homologous to *C. elegans* POT-1 and POT-2 proteins (Figure 5). The Q94E mutation has been found in individuals from families with high levels of melanoma and has also been identified as a recurrent somatic mutation that occurs during development of chronic lymphocytic leukemia [187,190]. Expression of several cancer-associated *POT1* variants leads to telomere elongation and sometimes to increased telomere fragility [188,191]. The long somatic telomeres caused by human germline *POT1* mutations could impede the ability of senescence to suppress tumor development but may also affect DNA damage signaling and recombination at telomeres [192,193].

As germline variants of human *POT1* act in a dominant manner to promote telomere elongation, they might be able to alter telomeric foci during gametogenesis. Germline *POT1* variants with relevance to cancer predisposition were identified because they cosegregate with individuals who present disease [178]. If multigenerational epigenetic inheritance is relevant to humans who possess germline *POT1* variants, then descendants who are wild-type for *POT1* might be affected. Indeed, some human pedigrees with *POT1* variants contain individuals who develop cancer but lack the *POT1* variant [186]. This could be explained by an inherited epigenetic effect of the *POT1* variant or by other genetic or environmental factors that contribute to tumor development. If some human *POT1* mutations alter levels of telomeric foci in children or grandchildren, then this might be linked to cancer development or to a phenotype that is distinct from telomere length and might be regulated by telomeric foci. Given that phenotypes relevant to altered levels of telomeric foci may not be obvious, it might be interesting to ask if human families transmit traits that affect multiple descendants of an individual in a manner that is consistent with transgenerational epigenetic inheritance.

Telomere length can be affected by environmental and lifestyle stresses, such as smoking [194], alcohol consumption [195], nutrition [196], and early life adversity [197]. It might be worth considering if an altered epigenetic state at telomeres is associated with health outcomes that can be disentangled from environmental covariables or altered telomere length. Genetic and environmental factors may be integrated in human germ cells to shape both the epigenetic state of telomeres and telomere length for chromosomes that are transmitted to future generations. If a heritable epigenetic state can occur at human telomeres, as observed for *C. elegans*, and if this state can be demonstrated to be relevant to human physiology [198], it is possible that telomere-specific epigenetic interventions could be developed to affect specific tissues or to reprogram human germ cells.

## Data Availability

Not applicable.
